# Completeness of T, N, M and stage grouping for all cancers in the Mallorca Cancer Registry

**DOI:** 10.1186/s12885-015-1849-x

**Published:** 2015-11-04

**Authors:** M. Ramos, P. Franch, M. Zaforteza, J. Artero, M. Durán

**Affiliations:** 1 Mallorca Cancer Registry, Public Health Department, Hospital Psiquiàtric 40, 07110 Palma, Balearic Islands Spain; 2 Hospital Son Espases Tumour Registry, Balearic Islands Health Service, Palma, Spain; 3 Hospital Manacor Tumour Registry, Balearic Islands Health Service, Manacor, Spain

**Keywords:** Cancer registry, Cancer staging, TNM, Completeness, Mediterranean Islands

## Abstract

**Background:**

TNM staging of cancer is used to establish the treatment and prognosis for cancer patients, and also allows the assessment of screening programmes and hospital performance. Collection of staging data is becoming a cornerstone for cancer registries. The objective of the study was to assess the completeness of T, N, M and stage grouping registration for all cancers in the Mallorca Cancer Registry in 2006–2008 and to explore differences in T, N, M and stage grouping completeness by site, gender, age and type of hospital.

**Methods:**

All invasive cancer cases during the period 2006–2008 were selected. DCO, as well as children’s cancers, CNS, unknown primary tumours and some haematological cases were excluded. T, N, M and stage grouping were collected separately and followed UICC (International Union Against Cancer) 7th edition guidelines. For T and N, we registered whether they were pathological or clinical.

**Results:**

Ten thousand two hundred fifty-seven cases were registered. After exclusions, the study was performed with 9283 cases; 39.4 % of whom were women and 60.6 % were men. T was obtained in 48.6 % cases, N in 36.5 %, M in 40 % and stage in 37.9 %. T and N were pathological in 71 % of cases. Stage completeness exceeded 50 % in lung, colon, ovary and oesophagus, although T also exceeded 50 % at other sites, including rectum, larynx, colon, breast, bladder and melanoma. No differences were found in TNM or stage completeness by gender. Completeness was lower in younger and older patients, and in cases diagnosed in private clinics.

**Conclusions:**

T, N, M and stage grouping data collection in population-based cancer registries is feasible and desirable.

## Background

The Tumour Node Metastasis (TNM) system is the most extended scheme of stage grouping in cancer [1], although there are still some cancers that cannot be classified within TNM system, such as children’s cancers, Central Nervous System (CNS) tumours and some haematological diseases. In others, lymphoma or myeloma for instance, stage grouping can be assessed but not T, N and M.

Stage grouping summarises the anatomical extension of a cancer at the moment of diagnosis and is based on three components: the T (primary tumour growth), the N (local lymph node involvement) and the M (distant metastasis). Stage grouping classifies cancers into: stage I (small or superficial localised cancer), stage II (large or deep localised cancer), stage III (regionally spread cancer) and stage IV (cancer with distant metastasis). In clinical practice, it is used to establish the treatment as well as the prognosis of each patient so it is important for both hospital clinicians and primary health care physicians. For population-based cancer registries, stage is becoming a cornerstone because it permits calculation of survival, assessment of the results of screening programmes and inter-hospital performance comparisons. However, only about 23 % of the cancer registries which contributed to the IX volume of Cancer Incidence in Five Continents, recorded stage grouping for all cancer topographies [2, 3]. The Danish Cancer Registry (DCR), the oldest in the world, is one of them [4].

The Mallorca Cancer Registry (MCR) covers the Spanish island of Mallorca, with around 800,000 inhabitants. It was created in 1989 by a group of clinicians, the so-called Grup d’Estudis del Càncer Colorectal. Not until 2008 was it integrated into the Public Health Department. MCR had started the registration of T, N and M and stage tentatively in 2000. Since 2006, it has become standard procedure for all cancer sites.

The objectives of this study are: 1) To assess the completeness of T, N and M and stage registration for all cancers in the MCR between 2006 and 2008, and 2) To explore differences in completeness by site, gender, age and type of hospital. Both objectives pursue the improvement the collection of T, N, M and stage grouping in the MCR, as well as its accuracy.

## Methods

MCR collects all invasive and in situ cancer cases at all topographies, plus uncertain and benign bladder and CNS tumour cases. Since 2000, registration of skin cancer cases other than melanoma, mainly squamous and basal cell carcinoma has been suspended because of the enormous workload it generates (around 40 % of all cancers).

### Dataset

All invasive cancer cases diagnosed in the period 2006 to 2008 were selected. Death Certificate Only (DCO) cases (cases identified only through the death certificate) as well as children’s cancers (0–14 years inclusive), CNS, unknown primary tumours (C26, C39, C48, C76 and C80) and some haematological cases (leukaemia and immunoproliferative, myeloproliferative and myelodysplastic syndromes) were excluded because they cannot be staged.

We included the following variables: gender; date of birth; type of hospital where the case was diagnosed (public versus private); date of diagnosis; topography and morphology according the International Classification of Diseases for Oncology (ICD-O) 3rd edition [5]; T, N, M and stage grouping according to guidelines in the Union for International Cancer Control (UICC) 7th edition [1]. We clustered some topographies and we identified some cancers through morphology (Table 1).

**Table 1:** ICD-O 3rd edition grouping used

Topographies	Cancers
C00-C14	Head and neck
C15	Oesophagus
C16	Stomach
C17	Small intestine
C18	Colon
C19-C21	Rectum
C22.0	Liver
C22.1, C24	Biliary tract
C23	Gallbladder
C25	Pancreas
C30, C31	Nasal cavity and sinuses
C32	Larynx
C34	Lung
C33, C37, C38	Other thoracic organs
C40, C41	Bone
C47, C48	Soft tissues
C50	Breast
C51	Vulva
C52	Vagina
C53	Cervix uteri
C54, C55	Uterus
C56	Ovary
C57	Other female genital organs
C60	Penis
C61	Prostate
C62	Testis
C63	Other male genital organs
C64	Kidney
C65-C68	Bladder and urinary tract
C69	Eye
C73	Thyroid gland
C74	Adrenal gland
Morphologies	Cancers
8720–8790	Melanoma
9590–9729	Lymphoma
9731–9734	Myeloma

T, N, M and stage grouping data were collected by two doctors and two nurses specifically trained from distinct sources: pathology reports; diagnostic imaging test reports such as computerised axial tomography or echo-endoscopy, and clinical records. Initially, we registered the information available in these reports, recording T, N, M and stage grouping separately. Nevertheless, when they were not available, but we could calculate it, we did. Indeed, for T and N components, we registered whether they were pathological (from pathology reports) or clinical (from radiology reports) according to UICC 7th edition rules [1]. For some gynaecological tumours, especially ovary and cervix, information on the stage grouping was available, but not the T, N and M components. Indeed, when M was 1, we assumed stage IV even in the absence of the T and N.

### Statistical analysis

A descriptive stratified analysis was performed using proportions and their confidence intervals at 95 % of T, N, M and stage grouping by topography or histology, gender, age and type of hospital. The SPSS 19th edition software package was used for statistical analysis.

This study is part of the quality assessments performed regularly in the MCR, so it has not been presented to any Ethical Committee. According to the Law 5/2003 of Health of the Balearic Islands and the Decree 6/2013 establishing the competency and organic structure of the Balearic Government, the Public Health Department has the competency to create and manage disease registries. MCR send regularly their data to the International Agency of Clinical Research (IACR) and to the European Network of Cancer Registries (ENCR). These data, in aggregate format, are openly available.

## Results

During the period 2006–2008, 10,257 cases of invasive cancer were registered in the MCR, of which 167 cases were excluded because they were DCO, 61 as they were in children, 165 because they were CNS, 221 cases due to unknown primary tumour and 427 as they were leukaemia or other haematological syndromes. Finally, the study was performed with 9283 cases.

In men, prostate cancer was the most frequent with 24.6 % of cases, followed by lung (20.9 %), bladder (11.3 %), and colon (10.2 %). In women, breast cancer was by far the most frequent with 34.2 % of cases, followed by colon (11.5 %), uterus (7.3 %) and lung (6.7 %) (Table 2).

**Table 2:** Completeness of TNM & stage registration in the Mallorca Cancer Registry (2006–2008)

Cancers	Number	T			N			M		Stage	
	cases	%	CI95 %	% p^a^	%	CI95 %	% p^a^	%	CI95 %	%	CI95 %
Head and neck	359	42.3	37.3–47.5	65.8	41.2	36.2–46.4	62.1	32.9	28.2–37.9	25.1	20.9–29.8
Oesophagus	103	44.7	35.4–54.3	23.9	50.5	41.0–59.9	19.2	54.4	44.8–63.7	53.4	43.8–62.7
Stomach	265	43.0	37.2–49.0	70.2	42.3	36.5–48.3	70.5	46.8	40.9–52.8	50.2	44.2–56.2
Small intestine	34	20.6	10.3–36.8	100.0	20.6	10.6–36.8	100.0	32.4	19.1–49.2	32.4	19.1–49.2
Colon	926	79.2	76.4–81.6	96.6	77.9	75.1–80.4	96.5	63.5	60.3–66.5	66.0	62.9–69.0
Rectum	493	72.8	68.7–76.5	68.8	70.4	66.2–74.2	68.9	61.9	57.5–66.0	50.3	45.9–54.7
Liver	217	1.8	0.7–4.6	100.0	0.0	0.0–1.7	0.0	5.5	3.2–9.4	6.0	3.5–10.0
Biliary tract	104	20.2	13.6–28.9	90.5	18.3	12.2–26.8	89.5	22.1	15.2–31.0	19.2	12.8–27.8
Gallbladder	52	30.8	19.9–44.3	93.7	23.1	13.7–36.1	83.3	38.5	26.5–52.0	26.9	23.1–48.2
Pancreas	224	23.2	18.2–29.2	26.9	22.8	17.8–28.7	29.4	47.8	41.3–54.3	48.7	42.2–55.2
Nasal cavity and sinuses	12	8.3	1.5–35.4	0.0	8.3	1.5–35.4	0.0	8.3	1.5–35.4	0.0	0.0–24.2
Larynx	168	54.2	46.6–61.5	81.3	50.0	42.5–57.5	71.4	43.5	36.2–51.0	38.1	31.1–45.6
Lung	1303	43.3	40.6–46.0	21.6	43.6	40.9–46.3	21.6	66.9	64.3–69.4	67.5	64.9–69.9
Other thoracic organs	34	20.6	10.3–36.8	57.1	14.7	6.4–30.1	40.0	23.5	12.4–40.0	23.5	12.4–40.0
Bone	33	12.2	4.8–27.3	75.0	12.1	4.8–27.3	75.0	21.2	10.7–37.7	6.1	1.7–19.6
Soft tissues	63	19.0	11.2–30.4	83.3	15.9	8.9–26.8	70.0	22.2	13.7–33.9	14.3	7.7–25.0
Breast	1169	65.3	62.5–67.9	87.7	60.7	57.8–63.4	86.9	48.7	45.8–51.5	44.4	41.6–47.3
Vulva	41	29.3	17.6–44.5	91.7	24.4	13.8–39.3	80.0	26.8	15.7–41.9	9.8	3.9–22.5
Vagina	7	0.0	0.0–35.4	0.0	14.3	2.6–51.3	0.0	0.0	0.0–35.4	14.3	2.6–51.3
Cervix uteri	141	35.5	28.0–43.6	80.0	28.4	21.6–36.3	82.5	24.8	18.4–32.6	41.1	33.3–49.4
Uterus	245	49.8	43.6–56.0	99.2	34.7	29.0–40.8	94.1	37.1	31.3–43.3	32.7	27.1–38.7
Ovary	162	17.3	12.2–23.8	96.4	9.9	6.2–15.4	93.7	22.8	17.0–29.9	53.1	45.4–60.6
Other female genital organs	6	16.7	3.0–56.3	100.0	16.7	3.0–56.3	100.0	0.0	0.0–39.0	0.0	0.0–39.0
Penis	28	53.6	35.8–70.5	93.3	17.9	7.9–35.6	40.0	28.6	15.2–47.1	14.3	5.7–31.5
Prostate	1294	34.0	31.5–36.6	79.5	13.1	11.4–15.1	75.9	25.7	23.4–28.2	11.2	9.6–13.0
Testis	77	53.2	42.2–64.0	100.0	15.6	9.1–25.3	25.0	42.9	32.4–54.0	18.2	11.1–28.2
Other male genital organs	3	0.0	0.0–56.1	0.0	0.0	0.0–56.1	0.0	0.0	0.0–56.1	0.0	0.0–56.1
Kidney	218	46.3	39.8–53.0	97.0	13.3	9.4–18.4	82.7	39.0	32.8–45.6	27.1	21.6–33.3
Bladder and urinary tract	710	77.9	74.7–80.8	35.8	13.2	10.9–15.9	70.2	17.3	14.7–20.3	12.7	10.4–15.3
Eye	20	0.0	0.0–16.1	0.0	0.0	0.0–16.1	0.0	5.0	0.9–23.6	20.0	8.1–41.6
Thyroid gland	114	37.7	29.4–46.9	97.7	15.8	10.2–23.6	83.3	21.1	14.6–29.4	3.5	1.4–8.7
Adrenal gland	7	42.9	15.8–74.9	66.6	28.6	8.2–64.1	50.0	57.1	25.0–84.2	42.9	15.8–75.0
Melanoma	286	65.0	58.6–70.8	100.0	24.8	19.7–30.7	91.4	32.1	26.4–38.3	12.4	8.8–17.2
Lymphoma	505	-			-		-	-		34.9	30.8–39.1
Myeloma	131	-			-		-	-		26.0	19.2–34.1
TOTAL	3514	48,6	47.5–49.6	70.6	36,5	35.6–37.5	70.9	40,6	39.6–41.6	37.9	36.9–38.8

^a^% T or N pathological

Distribution of cases by gender was as follows: 39.4 % in women and 60.6 % in men. Regarding age at diagnosis: 4.8 % were under 40 years old, 42.3 % were between 41 and 65, 38.7 % between 66 and 80 and 14.2 % older than 80. Three out of four patients (74.2 %) were treated at public hospitals, and the rest (25.8 %) at private clinics.

Stage grouping data was obtained in 3514 cases (37.9 %), T in 4508 cases (48.6 %), N in 3392 (36.5 %) and M in 3769 (40.6 %). T and N components were pathological in 70.6 % and 70.9 % of cases respectively. Distribution of T, N, M and stage grouping completeness by topography or histology is shown in Table 1. Stage grouping completeness exceeded 50 % in lung, colon, ovary and oesophagus; T completeness exceeded 50 % in colon, rectum, larynx, breast, penis, testis, bladder and urinary tract and melanoma; N completeness exceeded 50 % in colon, rectum and breast; and finally, M completeness exceeded 50 % in oesophagus, colon, rectum, lung and melanoma. T and N were mostly pathological in colon, gallbladder, larynx, breast, vulva, cervix uteri, uterus, ovary, penis, prostate, kidney and thyroid gland, and mostly clinical in stomach, pancreas, lung and bladder and urinary tract. In some cases, such as the ovary, T and N were recorded in less than 20 % of cases, although stage grouping was registered in more than half.

Distribution of T, N, M and stage grouping completeness by gender, age and type of hospital can be seen in Table 3. Differences by age and type of hospital were observed, but not by gender.

**Table 3:** Completeness of TNM & stage by sex, age and type of hospital in Mallorca Cancer Registry (2006–2008)^a^

		Number	T		N		M		Stage	
Variable	Categories		%	CI95 %	%	CI95 %	%	CI95 %	%	CI95 %
Sex	Women	3657	49.5	47.9–51.2	42.2	40.6–43.8	40.6	39.9–42.2	41.1	39.5–42.7
	Men	5626	47.9	46.6–49.2	32.9	31.6–34.1	40.6	39.3–41.2	35.7	34.5–37.0
Age	<40	447	44.1	39.5–48.7	32.0	27.8–36.4	32.9	28.7–33.0	32.9	28.7–37.4
	40–65	3926	51.6	50.0–53.1	40.8	39.3–42.3	44.3	42.8–45.9	41.0	39.4–42.5
	66–80	3590	49.5	47.9–51.1	36.0	34.4–37.5	39.8	38.2–41.4	37.2	32.6–35.7
	>80	1320	38.6	36.0–41.2	27.0	24.6–29.4	34.2	31.7–36.8	32.2	29.7–34.8
Type of hospital	Public	6889	54.7	53.5–55.9	41.1	38.9–42.2	47.4	46.3–48.6	44.2	43.0–45.4
	Private	2394	30.8	29.0–32.7	23.6	21.9–25.3	20.9	19.3–22.6	19.5	18.0–21.1

^a^Percentages

## Discussion

We hope that this paper will be of interest not only to cancer-registry staff, but also to hospital clinicians and primary health care physicians, as they would benefit most from an optimal registration of T, N, M and stage grouping, and are well placed to contribute to building a comprehensive population-based cancer register.

We obtained stage grouping data in almost one in two cases. It is clear that this percentage is not high enough. But looking through the results, we realise that we have been too conservative in N and M assignment. First, according to the 7th edition of the IUCC TNM guide [1], the use of X for the M category is considered to be inappropriate as clinical assessment of metastasis can be based on physical examination alone. Following this rule, the percentage of M obtained in our series should be 100 % rather than 40 %, so we could assume the remaining 60 % to be M0, especially if the patient is alive. Furthermore, in daily practice, oncologists and other clinicians make assumptions to complete the TNM components and make treatment decisions. Cancer registries could do the same, taking advantage of the fact that T, N, M and stage data are collected retrospectively. For instance, looking at our data for bladder and urinary tract cancer, we only obtained 5.9 % of stage grouping, but 77.7 % of the T component, while in the DCR they obtained 44.1 % of stage grouping, but only 61.8 % of T [6]. We believe that it could be assumed that all T1 cases are stage 1, even if the N component has not been verified, especially if they are asymptomatic and alive 5 years after the diagnosis.

To reduce the percentage of missing values, an alternative is to use the Summary Stage 2000 proposed by the American Surveillance, Epidemiology and End Results Program (SEER), a simplified scheme of staging grouping in: in situ, localised, locally extended and disseminated cancers, as the DCR did, assuming a relatively small loss of information compared to the stage grouping [7–10]. In our opinion, it is better to keep using T, N, M and stage grouping, trying to reduce the percentage of unknown stage cases and using multiple imputation method to deal with missing values, as they have shown to provide accurate estimates [11, 12].

Completeness of T, N, M and stage grouping was similar in men and women. Regarding age, we did not observe a decline with age as seen in the DCR or the SEER registries [6–10, 13], but a lower completeness in young adults and elderly. We believe that there are different explanations for both age groups. In patients under 40 years old, perhaps it is due to a predominance of cancers that cannot be staged with TNM, as it happen with childhood cases. On the other hand, in patients over 80, we found lower T, N and M, but a similar percentage of stage grouping, probably due to less aggressive treatments in elderly people [14, 15]. Finally, as expected, considerably less T, N, M and stage grouping data have been found for patients diagnosed in private clinics. This is related to the absence or the inaccessibility of clinical records in these centres. Improvements in this area would be beneficial.

Apart from completeness, the accuracy of T, N, M and stage grouping in cancer registries should also be assessed, as New Zealand Cancer Registry is doing [14]. In our case, the benchmark should be the revision of clinical records by an oncologist. It would be interesting also to compare the accuracy of T, N, M and stage grouping between automatic data collection systems, such as the DCR, and registries using manual collection such as the MCR. It has to be admitted that collection of T, N, M and stage grouping using our method involves the review of almost all clinical records, which is laborious but feasible thanks to the availability of electronic clinical records. Furthermore, a high degree of accuracy can be expected, since multiple sources are used.

The current TNM System already does include some symptoms and molecular patterns. Future editions will almost certainly include more, as some authors claim [16]. Nevertheless, in population-based cancer registries, which prioritise quality over amount of information collected, adding new variables to their dataset has to be carefully considered because each new variable can dramatically increase the workload involved. Obtaining feedback from oncologists and other clinicians would be appropriate in this regard.

## Conclusions

In conclusion, collection of T, N, M and stage grouping data in population-based cancer registries is feasible and desirable, as stage is the main prognostic factor in many cancers. An international agreement on recommended T, N and M assumptions for missing data is proposed in addition to another for the eventual inclusion of new variables for stage grouping.

## Abbreviations

CNS: Central Nervous System
DCO: Death Certificate Only case: Case indentified only through the death certificate
DCR: Danish Cancer Registry
ICD-O: International Classification of Diseases for Oncology
MCR: Mallorca Cancer Registry
M: Metastasis
N: Local lymph node involvement
SEER: Surveillance, Epidemiology and End Results Program (US)
T: Primary tumour growth
TNM: Tumour Node Metastasis System
UICC: International Union Against Cancer
